# Increased reactive oxygen species levels cause ER stress and cytotoxicity in andrographolide treated colon cancer cells

**DOI:** 10.18632/oncotarget.15393

**Published:** 2017-02-16

**Authors:** Aditi Banerjee, Vivekjyoti Banerjee, Steven Czinn, Thomas Blanchard

**Affiliations:** ^1^ Department of Pediatrics, University of Maryland School of Medicine, Baltimore, Maryland, U.S.A

**Keywords:** andrographolide, chemotherapy, reactive oxygen species, endoplasmic reticulum stress, unfolded protein response

## Abstract

Chemotherapy continues to play an essential role in the management of many cancers including colon cancer, the third leading cause of death due to cancer in the United States. Many naturally occurring plant compounds have been demonstrated to possess anti-cancer cell activity and have the potential to supplement existing chemotherapy strategies. The plant metabolite andrographolide induces cell death in cancer cells and apoptosis is dependent upon the induction of endoplasmic reticulum stress (ER stress) leading to the unfolded protein response (UPR). The goal of the present study was to determine the mechanism by which andrographolide induces ER stress and to further evaluate its role in promoting cell death pathways. The T84 and COLO 205 cancer cell lines were used to demonstrate that andrographolide induces increased ROS levels, corresponding anti-oxidant response molecules, and reduced mitochondrial membrane potential. No increases in ROS levels were detected in control colon fibroblast cells. Andrographolide-induced cell death, UPR signaling, and CHOP, Bax, and caspase 3 apoptosis elements were all inhibited in the presence of the ROS scavenger NAC. Additionally, andrographolide-induced suppression of cyclins B1 and D1 were also reversed in the presence of NAC. Finally, Akt phosphorylation and phospho-mTOR levels that are normally suppressed by andrographolide were also expressed at normal levels in the absence of ROS. These data demonstrate that andrographolide induces ER stress leading to apoptosis through the induction of ROS and that elevated ROS also play an important role in down-regulating cell cycle progression and cell survival pathways as well.

## INTRODUCTION

The phytochemical andrographolide (Andro), is a bicyclic diterpenoid lactone purified from *Andrographis paniculata*, a herb widely used throughout Asia as a traditional remedy for numerous maladies. It has been used extensively to treat both infectious and chronic diseases [1], and both *in vitro* and *in vivo* experimental models provide detailed evidence that Andro possesses potent anti-inflammatory properties [2]. Andrographolide has also been demonstrated to possess multifaceted anticancer cell activity and has been tested against human cells from breast cancer [3, 4], lung cancer [5, 6], leukemia [7], colon cancer [8, 9], liver cancer [10, 11], prostate cancer [12, 13], and others. These models have been used to determine that Andro activates pro-apoptosis pathways and induces cell cycle arrest at both the G1/S and G2/M phases. Studies *in vivo* employing murine xenograft models of human cancers have yielded positive results when treated with Andro demonstrating delayed tumor growth when applied either alone or in combination with other chemicals [14–17]. Although many studies describe the various signaling events leading to apoptosis and measure the factors that regulate cell cycle progression in the context of Andro treatment, little is known about the early cellular events following Andro treatment that lead to these events.

We recently reported that Andro-induced cell death occurs via ER stress in colon cancer cells as demonstrated by blocking the unfolded protein response (UPR) [18]. While ER stress can initiate downstream signaling leading to apoptosis via the IRE-1, PERK, and ATF6 ER membrane proteins, we observed that Andro-induced cell cytotoxicity occurred primarily through IRE-1 activity as shown by over expression of IRE-1 as well as depletion of IRE-1 with siRNA. The ER stress response is best understood in the context of an accumulation of unfolded or incorrectly folded proteins [19]. The cell responds to such alterations through the UPR in which proteins such as GRP78, IRE-1, PERK, and ATF6 transmit signals to activate mechanisms to ameliorate the accumulation of the altered proteins. When ER stress becomes irreversible, these same pathways will promote apoptosis to eliminate the cells.

Many factors can contribute to the induction of ER stress and the UPR including over-expression of proteins beyond the capacity of the ER to correctly fold them, inhibition of glycosyation [20], ER Ca^2+^ depletion, and oxidative stress among others. We now report that Andro induced ER stress/UPR leading to apoptosis is dependent upon the induction of oxidative stress. Andro induces reactive oxygen species (ROS) along with expression of multiple antioxidant response genes. Inhibition of ROS significantly reduces expression of UPR proteins as well as cell death and proapoptosis pathways. We also report that in addition to inducing apoptosis via the UPR, Andro blocks Akt phosphorylation resulting in decreased levels of mTOR, and suppresses Cyclins B1 and D1 of the cell cycle progression pathway. Scavenging of Andro-induced ROS blocked these activities. These data provide additional insight into the anticancer cell activity of Andro.

## RESULTS

### Andrographolide selectively inhibits colon cancer cells

**Figure 1:**
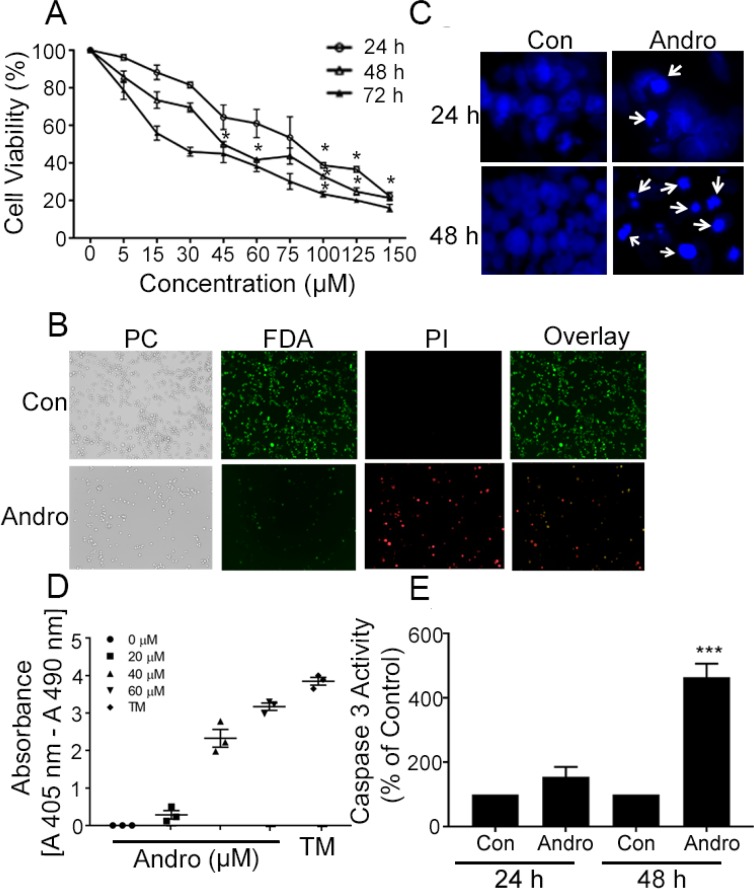
Andrographolide suppresses cell proliferation and cell survival in COLO 205 cells (**A**) Cells were treated with Andro for 24, 48 and 72 h and cell viability was quantified by MTT assay. (**B**) Fluorescence microscopy images showing the viability of COLO 205 cells cultured *in vitro* with or without Andro (45 μM) (left to right: phase contrast (PC) image, FDA stain, PI stain, overlay of FDA and PI stain). (**C**) Cells were treated with Andro IC_50_ dose (45 μM) for either 24 h (upper panels) or 48 h (lower panels) and stained with DAPI. Apoptotic cells were identified by condensation and fragmentation (arrows) of nuclei using inverted fluorescence microscope. (**D**) Detection of nucleosomes in cytoplasmic fractions at increasing doses of Andro and TM. 10^4^ cells were treated with or without Andro (20, 40, or 60 μM) or TM (1μg/ml) for 48 h at 37°C. Cell lysates (20 μl) were analyzed in the ELISA. (**E**) Caspase 3 activity was evaluated by Caspase-3 colorimetric activity assay kit as described. Experiments were performed two times (D and E) or three times (A, B and C). (*P < 0.05, ***P < 0.001).

The MTT assay was used to evaluate the effects of Andro on colon cancer COLO 205 cell numbers when treated for up to 72 h. There was a dose and time dependent inhibition of cell viability (Figure 1A) The IC_50_ at 24, 48 and 72 h was determined to be 80, 45, and 26 μM respectively. Treatment of normal colon epithelial cells with the same concentration of Andro had little effect on cell numbers which only dropped below 80% at the highest dose tested (Supplementary Figure 1). These data suggest that Andro selectively inhibits colon cancer cells but not normal colon cells. These results were consistent with our previous report utilizing T84 and HCT 116 colon cancer cell lines to test Andro activity and the IC_50_ (45 μM) at 48 h was used for subsequent assays. FDA-PI double staining of Andro treated COLO 205 cells revealed the incorporation of less FDA and increased PI staining indicating increased cell death relative to untreated control cells (Figure 1B). To determine whether the Andro associated decreased viability was due to the induction of apoptosis, nuclear morphology was examined by microscopy using DAPI staining. Treatment of COLO 205 cells with Andro (45 μM) for 24 h and 48 h revealed several apoptotic morphological features including apoptotic bodies, cell shrinkage, and chromatin condensation not observed in control cells, most evident at 48 h (Figure 1C). The degree of apoptosis was assessed further by quantitative ELISA to measure apoptotic nucleosomes. Nucleosomes present in cell lysates following 48 h treatment with Andro increased in a dose dependent manner (Figure 1D). Additionally, caspase-3 activity was also found to be increased significantly in Andro-treated COLO 205 cells at 48 h (*P* < 0.001) (Figure 1E).

### Andrographolide treatment of colon cancer cells is associated with ROS generation and loss of mitochondrial membrane potential

**Figure 2:**
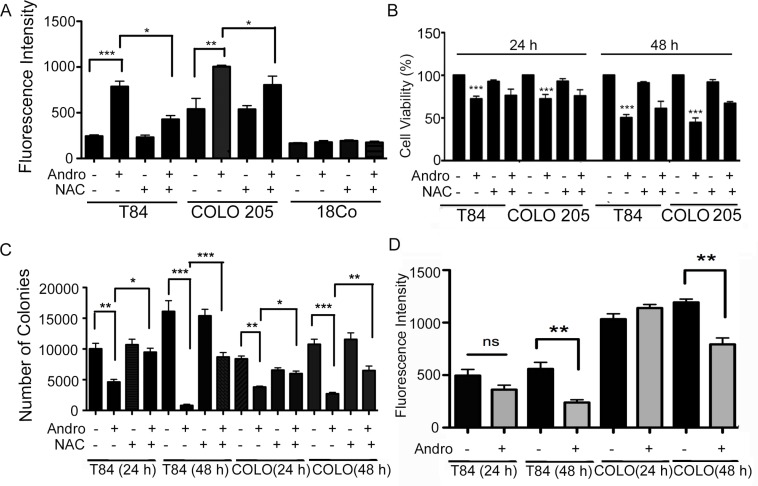
Andrographolide induces ROS generation in colon cancer cells (**A**) The effects of Andro treatment on ROS generation. T84, COLO 205, and normal fibroblast cells (18Co) were treated with Andro IC_50_ (45 μM) for 24 h and cellular ROS levels were determined by measurement of fluorescent DCF. ROS inhibition was performed by pre-treatment of cells with 20 mM NAC for 1 h prior to Andro treatment. (**B**) The effects of ROS on cell viability in Andro-treated colon cancer cells were assessed by MTT assay at 24 h and 48 h in the presence of absence of NAC. Absorbance was read at 570 nm with averages from triplicate wells. (**C**) For quantification of clonogenicity Andro treated T84 and COLO 205 cells were examined by colony formation assay for 24 h and 48 h and visualized by staining with crystal violet. (**D**) T84 and COLO 205 cells were treated with or without Andro for 24 h and 48 h and then incubated with TMRE. Experiments were performed two times (A and D) or three times (B and C). (*P < 0.05, **P < 0.01, ***P < 0.001).

Andrographolide induced cell death in Colon T84 and HCT 116 cancer cell lines has been shown to be dependent upon ER stress, specifically through IRE-1 signaling leading to CHOP expression and the induction of apoptosis [18]. The same events were observed in Andro-treated COLO 205 cells as GRP78, IRE-1, and CHOP expression were significantly increased at both 24 and 48 h and this activity could be inhibited with the ER stress inhibitor 4-PBA (Supplementary Figure 2). Many factors can induce ER stress including oxidative stress, and the anti-cancer effects of certain plant metabolites have been shown to trigger ROS generation [21]. We therefore investigated the role of ROS in Andro anticancer activity. Colon cancer cell lines were treated with Andro for 24 and 48 h and the amount of intracellular ROS was assessed using a dichlorofluorescein (DCF) based assay. Andrographolide increased ROS generation in both T84 and COLO 205 cells compared with untreated control cells by 24 h (*P* < 0.001 and *P* < 0.01 respectively), whereas no increase was detected in human fibroblast 18Co cells (Figure 2A). Similar results were observed at 48 h (data not shown). Pretreatment of cells with the ROS inhibitor N-acetyl-L-cysteine (NAC) 1 h prior to Andro treatment resulted in a significant decrease in ROS (*p* < 0.05) in both colon cancer cell lines compared to cells treated with Andro alone. We next examined the role of intracellular ROS levels in the growth inhibition of colon cancer cells during Andro treatment. The ROS scavenger inhibitor NAC (20 mM) was added 1 h before Andro administration and the cells were evaluated for viability by the MTT assay. The NAC pretreatment significantly inhibited Andro-induced growth inhibition in T84 and COLO 205 cells at both 24 and 48 h (*P* < 0.001) (Figure 2B). Similar results were observed in a clonogenic assay where NAC pretreatment significantly increased cell viability in Andro treated cells (Figure 2C).

Generation of ROS is associated with disruption of mitochondrial membrane potential (MMP) [22]. Therefore, we investigated the effect of Andro on the integrity of the mitochondria by staining the cells with a cell permeant dye that readily accumulates in active mitochondria due to their relative negative charge. As shown in Figure 2D, incubation of T84 and COLO 205 cells with Andro caused a significant decrease of MMP at 48 h (*P* < 0.01).

### Andrographolide induces expression of antioxidant genes

**Figure 3:**
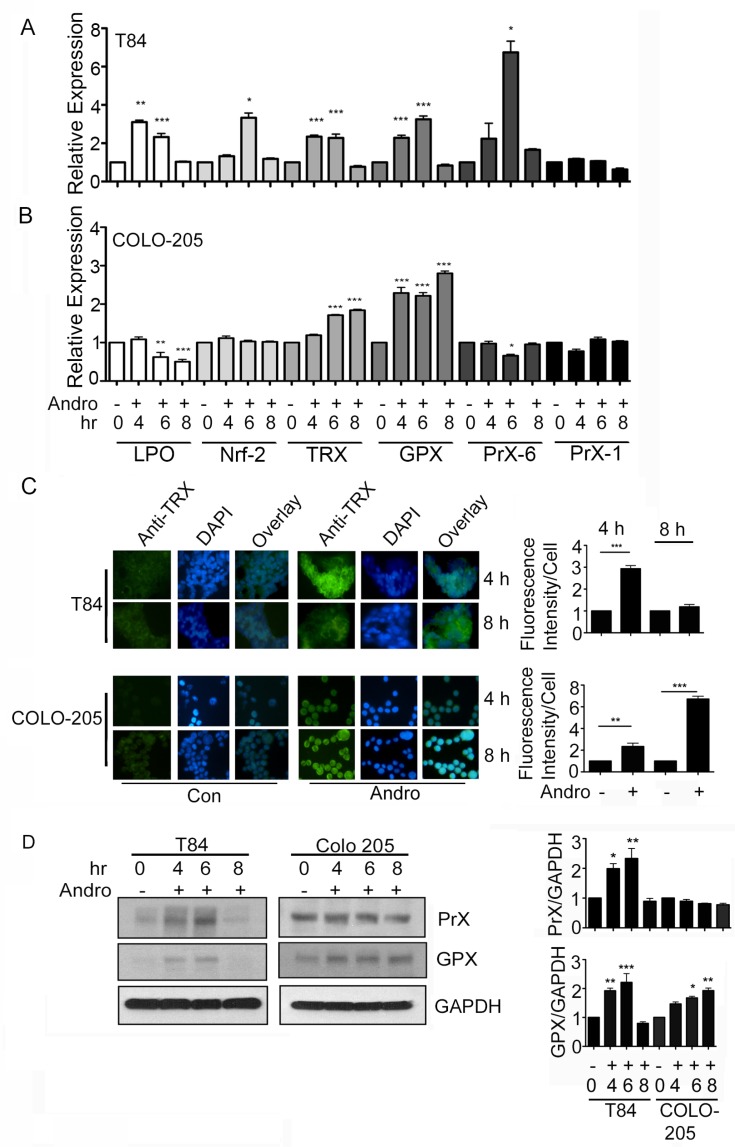
Andrographolide induces anti-oxidant gene expression T84 and COLO 205 cells were treated with or without Andro at IC_50_ (45 μM) for 4, 6, or 8 h and the transcriptional level of expression for anti-oxidant genes (LPO, Nrf-2, Trx, GPx, Prx-6) were determined by qRT-PCR for (**A**) T84 cells and (**B**) COLO 205 cells. Bar graphs show quantitative results normalized to GAPDH mRNA levels. Results are from three independent experiments. (**C**) T84 and COLO 205 cells were grown on coverslips and treated with Andro. TRX expression was evaluated by immunofluorescent staining. Nuclei were stained using DAPI and cells were examined by fluorescence microscopy. Fluoresence intensity was determined and compared with untreated (Con) T84 and COLO 205 cells. (**D**) T84 and COLO 205 cells were treated with Andro for 4 h, 6 h and 8 h. Cells were lysed and protein expression was determined by immunoblotting for PrX, GPX and GAPDH. Densitometry analysis was performed and normalized with GAPDH. Statistical significance was determined using one way-ANOVA followed by post hoc Tukey's test. (*P < 0.05, **P < 0.01, ***P < 0.001).

The up regulation of antioxidant genes can protect cells from injury due to oxidative stress. We measured transcript expression of six antioxidant genes as additional evidence that Andro induces significant ROS production. RNA was purified from T84 and COLO 205 cells at 4, 6, and 8 h after the addition of Andro and evaluated by real time PCR. Protein expression was measured by immunoblot or immunofluorescence. The gene expression of LPO, Nrf2, TRX, GPX, and PrX-6 in T84 cells were all significantly increased by 6 h (Figure 3A). However, all gene expression returned to base level by 8 h. Interestingly Andro had no impact on the expression of PrX-1. The response in COLO 205 cells was observed to be much narrower with only TRX and GPX increasing due to treatment with Andro (Figure 3B; *P* < 0.001). In the case of GPX, a significant increase was seen by 4 h and remained high even at 8 h. COLO 205 cells were also evaluated for antioxidant gene expression at 48 h of Andro treatment in the presence or absence of NAC (data not shown). The gene expression of LPO, Trx and GPx was increased greater than 1.5 fold compared to untreated cells and this activity was significantly reduced in the presence of NAC (data not shown). Trx protein was significantly elevated in Andro treated T84 at 4 h and in COLO 205 cells at 4 and 8 as demonstrated by immunofluorescence (Figure 3C). Immunoblot for Prx and GPx protein demonstrated transient expression in T84 cells that returned to background by 8 h (Figure 3D). GPx was also observed in COLO 205 cells and continued to be expressed at 8 h.

### Andrographolide induced ER stress and proapoptosis signaling are ROS dependent

**Figure 4:**
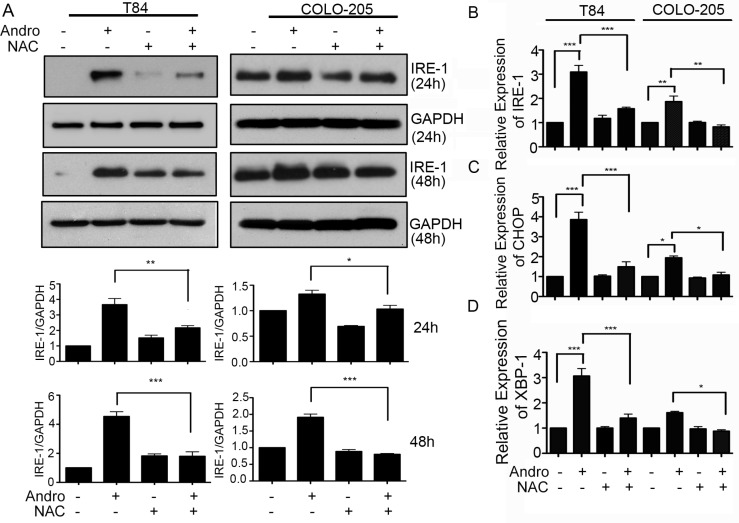
Andrographolide mediated ER stress and apoptosis signaling are dependent on increased ROS (**A**) T84 and COLO 205 cells were pre-treated with or without NAC followed by Andro IC_50_ (45 μM) for 24 h or 48 h. Cells were lysed and protein expression for IRE-1 and GAPDH were evaluated by immunoblot. Densitometry analysis was performed and normalized with GAPDH. Cells were also evaluated for mRNA expression by qRT-PCR for (**B**) IRE-1 and pro-apoptosis downstream signaling proteins, (**C**) CHOP, and (**D**) XBP-1. Results shown are from three independent experiments (*P < 0.05, **P < 0.01, ***P < 0.001).

Colon cancer cells were treated with Andro in the presence or absence of the ROS scavenger NAC to determine the relationship between ROS and ER stress. Immunoblot analysis for the UPR protein IRE-1 demonstrated a significant decrease in cells pretreated with NAC at both 24 and 48 h, and for both T84 and COLO205 cells with expression returning to those observed in the absence of Andro exposure (Figure 4A). Similar results were achieved when assessing gene expression data from cells harvested at 48 h Andro treatment which revealed that IRE-1 levels were significantly reduced when pretreated with NAC compared to those cells receiving Andro alone for both T84 and COLO 205 cells (*P* < 0.001; *P* < 0.01 Figure 4B). The proapoptosis signaling proteins CHOP and XBP-1 were also reduced in the presence of NAC in both colon cancer cells compared with the NAC and Andro treated cells (Figure 4C and 4D respectively). These data support a model in which Andro promotes ER stress leading to apoptosis signaling via the induction of ROS. These data are consistent with our previous report documenting ER stress mediated signaling by IRE-1 as quantification of gene expression for both PERK and ATF6 revealed no increase, and the anti-apoptosis molecule Bcl-2 showed no increase as well (Supplementary Figure 3).

### Andrographolide induced G1/S and G2/M cell cycle arrest and apoptosis in colon cancer cells is ROS dependent

**Figure 5:**
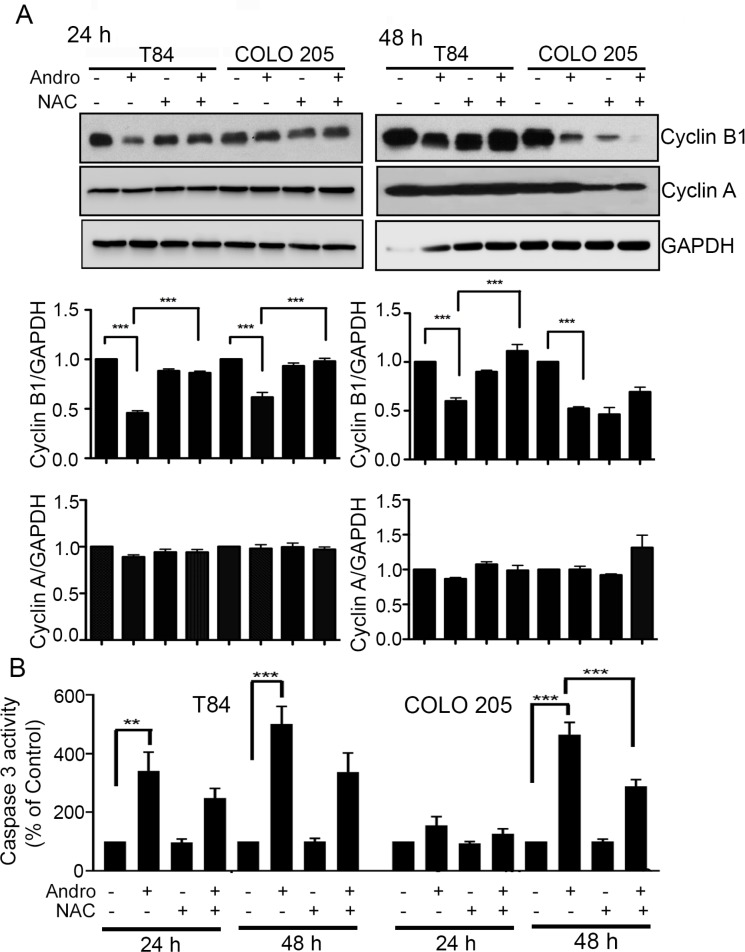
Andrographolide induced cell cycle arrest and apoptosis in colon cancer cells is ROS dependent (**A**) T84 and COLO 205 cells were pretreated with or without NAC followed by Andro IC_50_ (45 μM) for 24 h and 48 h. Cell lysates were analyzed by immunoblot and quantified by densitometry for expression of Cyclin B1 and cyclin A. Expression is normalized against GAPDH expression. (**B**) T84 and COLO 205 cells were pretreated with or without NAC followed by Andro IC_50_ for 24 h and 48 h. Cells were then lysed and incubated with caspase 3 substrate and activity was determined by colorimetric assay at OD_405nm_. (**P < 0.01, ***P < 0.001).

The cell cytotoxicity activity of Andro is largely the result of apoptosis and cell cycle arrest. We therefore investigated the degree to which these two Andro induced mechanisms are dependent upon oxidative stress by evaluating the effect of Andro in the presence and absence of NAC. Immunoblot analysis was performed on Cyclin A and Cyclin B1, cell cycle regulatory proteins that play important roles in regulating cell cycle progression. Figure 5A shows Andro could effectively suppress Cyclin B1 expression (*P* < 0.001) at both 24 h and 48 h compared to untreated control cells in both colon cancer cell lines. Pretreatment with NAC however resulted in Cyclin B1 expression being significantly up regulated (*P* < 0.001) compared with cells treated with Andro alone with the exception of COLO 205 cells at 48 h. No Andro associated increase was observed for Cyclin A. Additional analysis by real time PCR for gene expression of Cyclin D1 demonstrated a significant decrease (*P* < 0.001) in Andro treated in COLO 205 cells compared to control cells (Supplementary Figure 4). Cyclin D1 expression was observed to increase significantly and return to the levels observed in control cells when pretreated with NAC and exposed to Andro (*p* < 0.01). When caspase 3 activation was measured as an indicator for apoptosis, we observed caspase 3 activation with Andro treatment at 24 h and 48 h (*P* < 0.001) for T84 cells while a significant activation was observed in COLO 205 cells only at 48 h (*P* < 0.001). Moreover, pretreatment with NAC completely halted Andro induced caspase 3 activation in T84 cells at 24 h (Figure 5B). No significant induction of caspase activity was observed when normal fibroblast cells were treated with Andro (Data not shown).

### Andrographolide induced oxidative stress inhibits the Akt/mTOR cell survival signaling

**Figure 6:**
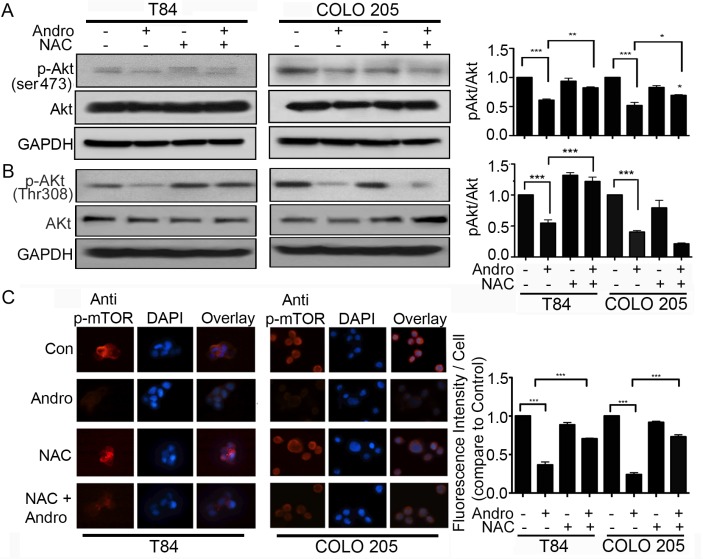
Andrographolide induced oxidative stress inhibits the Akt/mTOR cell survival signaling pathway (**A–B**) Cell lysates from T84 and COLO 205 cells pretreated with or without NAC and then treated with Andro IC_50_ (45 μM) for 48 h were analyzed by immunoblot for expression of phospho-Akt (ser473 and Thr 308), total Akt and GAPDH and quantified by densitometry. (**C**) T84 and COLO 205 cells were grown on coverslips and pretreated with or without NAC, followed by Andro IC_50_ for 48 h and then phospho-mTOR expression was evaluated by immunofluorescent staining. Nuclei were stained using DAPI and cells were examined by fluorescence microscopy. Fluorescence intensity was determined and compared with untreated T84 and COLO 205 cells.

Andrographolide inhibits cell survival. We therefore examined the possibility that Andro also induces cell death through regulating the Akt /mTOR signaling, a major pathway in the growth and survival of cancer cells. We observed a significant Andro-induced down regulation of phospho-Akt (ser 473) and phospho-Akt (Thr 308) expression in both colon cancer cell lines at 24 h and 48 h by immunoblot (Figure 6A and 6B respectively). Pretreatment of the cells with NAC before Andro exposure resulted in a significant increase in phospho-Akt (ser 473) in both T84 cells and COLO 205 cells (*P* < 0.01 and *P* < 0.05, respectively). An increase in phospho-Akt (Thr 308) was only observed in T84 cells (P < 0.001). Analysis of phospho-mTOR levels in Andro treated cells by immunofluorescence was consistent with phospho-Akt results (Figure 6C). There was a highly significant reduction in phospho-mTOR in both cell lines when treated with Andro (*P* < 0.001) but blocking ROS with NAC during Andro treatment significantly elevated phospho-mTOR expression almost to control levels (*P* < 0.001).

## DISCUSSION

The present study demonstrates the importance of ROS in Andro-induced anti-cancer cell activities. The ability of Andro to induce apoptosis and cell cycle arrest in multiple types of cancer is well documented [2], and we have recently established that Andro induced apoptosis is dependent upon the induction of ER stress leading to signaling through the UPR IRE-1 protein [18]. There are multiple molecular events that can result in ER stress including oxidative stress. Our data show that treatment of colon cancer cells with Andro induces ROS production in addition to LPO, TRX, and GPX antioxidant response genes. Pretreatment of cells with the ROS scavenger NAC effectively inhibited Andro induced IRE-1expression, proapoptotic signaling, caspase 3 activity and cell death.

The apoptosis events we observed were in accordance with the early induction of ROS. We observed elevated ROS at 24 hours and although ROS levels were not determined at earlier time points, TRX and GPX antioxidant response genes were significantly elevated between four and eight hours. This is consistent with the elevated caspase 3, CHOP, and XBP-1 expression and decreased Cyclin B1 and D1 expression we documented at 24 hours as well as the beginning of nuclear fragmentation. Fragmentation increased by 48 hours at which time we also observed increased nucleosome accumulation. ROS and anti-oxidant response genes also continued to be upregulated. Several laboratories have described an Andro induced increase in ROS that is important for cell cytotoxicity in treated cancer cells [23–28]. Additionally, other plant derived molecules with anti-cancer cell activity have been shown to induce ROS that is important for apoptosis signaling [21, 29]. Our data demonstrate that ROS production occurs upstream of ER stress and that the UPR is a crucial intermediate event in the ROS mediated anticancer cell activities of Andro.

It is noteworthy that ROS is important for two aspects of Andro mediated cell cytotoxicity, apoptosis and cell cycle arrest. While the pathways that lead to apoptosis can be established, the pathway leading to cell cycle arrest is less clear. The present study is consistent with several others in demonstrating down regulation of the Akt/mTOR survival pathway [15, 30–32]. Blocking this pathway can contribute to cell cycle arrest. However, the source of this down regulation has not been established. Activation of ERK has been demonstrated in some models to block Akt activation [33], but most Andro related studies report suppression or a lack of ERK activation [34–37]. There are notable exceptions where Andro associated cancer cell death was due in part to increased phopsho-ERK [26]. Alternatively, Andro may be inhibiting an event upstream of Akt activation.

In addition to cell cycle control, Akt/mTOR signaling also regulates autophagy, a process for sequestering cytosolic components and organelles for degradation by lysosomes. [38] Autophagy can also be play a role cell death, and suppression of mTOR signaling can lead to cancer cell death [39, 40]. Similar to some other natural compounds, Andro has been demonstrated to induce autophagic cell death in several different types of cancer [30, 31, 41]. Our data demonstrating the suppression of Akt and mTOR activation in colon cancer cells are consistent with suppression of the cell survival pathway and may support autophagic cell death. The present study examined the role of ROS in the context of the recently described ER-stress/apoptosis pathway [18]. The reduced ER stress, apoptosis, and cell cycle arrest markers we observed when ROS were scavenged does not preclude the involvement of autophagy in andro-induced cell death, or the contribution of ROS towards promoting the autophagic cell death. However, unlike other cancer cell systems of andro-induced autophagic cell death in which apoptosis cell death was demonstrated to play no role, our previous studies and those of others, along with the present study demonstrate a significant role of apoptosis in andro-mediated cell death of colon cancer cells. The importance of autophagy is this system will be the subject of future investigation.

It will also be important in defining the mechanism of Andro induced cell death, to determine the source of the ROS that drives the ER stress response. While there are anti-inflammation models in which Andro is shown to suppress ROS generation by NADPH oxidase there are other models in which induced ROS levels are crucial for the apoptosis and cell cycle arrest achieved with Andro in cancer cell killing [24–28, 42]. It is likely that the ability of Andro to induce ROS is cell type dependent as Andro seems to have distinct mechanisms of action when used to treat cancer and noncancerous cells. These studies also indicate that Andro has molecular activity upstream of ER stress since, regardless of the source of ROS, Andro must induce molecular events that favor ROS production. It is noteworthy that a previous study documenting the importance of Andro mediated ROS in cell cytotoxicity reported the phosphorylation of the p47 NADPH oxidase subunit as an important event [43]. The authors hypothesized a model in which Andro activates sphingomyelinase in the cell membrane to generate ceramide which then activates membrane NADPH oxidase via phosphorylation of the p47 subunit.

The initial events of Andro molecular activity when promoting cell death in cancer cells remain ill defined despite extensive research on the effector mechanisms leading to cytotoxicity. Defining a crucial role for increased ROS production in generating ER stress leading to apoptosis, or in regulating the pathways that control cell cycle progression provides insight into initial activity of Andro that may ultimately help identify targets for improved anti-cancer therapy. While it is possible that Andro activity occurs through multiple early pathways or receptors, elucidation of the molecular events that occur when Andro first contacts a cancer cell leading to ROS production will help clarify an important aspect of Andro activity.

## MATERIALS AND METHODS

### Cell culture and andrographolide treatment

The T84 (ATCC^®^ CCL-248™) and COLO 205 (ATCC^®^ CCL-222^™^) human colon cancer cell lines, normal colon epithelial cells FHC (ATCC^®^CRL-1831)^TM^ and the CCD-18Co human fibroblast cell line (ATCC^®^ CRL-1459^™^), were purchased from the American Type Culture Collection (Manassas, VA) and grown in the recommended complete tissue culture media. Cells were grown until approximately 75% confluent and then the media was replaced with fresh serum-free media containing 1/3 the concentration of antibiotic-antifungal solution (100X stock solution used at 0.33X) for 3 h. The media was then replaced with media containing 2% FBS with Andro (Sigma Aldrich, St. Louis, MO) at IC_50_ for 24 and 48 h. Stock Andro was prepared in DMSO and control wells received DMSO at a final concentration of 0.01%. Tunicamycin (2 μg/ml) was used as a positive control for ER stress induction. The inhibitor 4-PBA (100 mM) was used to block ER stress and NAC (20 mM) was used to block ROS. When these inhibitors were used, they were added 1 h before Andro administration.

### Cell viability assays

Cell viability was assessed using the MTT assay (Sigma Aldrich) as previously described [18]. Briefly, cells were treated with the indicated concentrations of Andro in 96-well plates for indicated time points followed by the addition of MTT solution to the cells (2.5 mg/ml final concentration). Plates were incubated at 37^°^C for 4 h and followed by the addition of dimethyl sulfoxide (DMSO). Optical densities (OD_570 nm_) were determined on a microplate reader (Model 550, Bio-Rad, USA). Cell viability was also determined for some experiments by fluorescein diacetate (FDA; F7378, Sigma) /propidium iodide (PI; P4170, Sigma) staining according to manufacturer's instructions (Ibidi, Madison, WI). Images were taken using afluorescence microscope.

### Clonogenic assay

Cells seeded at 5 × 10^5^/well in 6 well plates were grown for 24 h, washed, and then treated with Andro for 24 and 48 h. Cells were then trypsinized and seeded at 5 × 10^3^ cells/well in 6 well plates for 14 days. Cells were fixed with 10% buffered formalin for 15 min, and then stained with 2 ml 0.01% (w/v) crystal violet for 30 min. Wells were washed with dH_2_O, air dried, and colonies were counted manually by examination with an inverted microscope.

### DAPI staining

Cells were evaluated by staining with DAPI (Vector Laboratories; Burlingame, CA) as previously described [44].

### DCFDA cellular ROS detection assay

A DCFDA cellular ROS detection assay (#ab113851, Abcam PLC, Cambridge, MA) was used to measure hydroxyl, peroxyl and other ROS activity within the cell. T75 flasks were seeded with 4 × 10^6^ cells and grown until 75% confluent. Cells were then treated with Andro in the presence or absence of NAC as described above for 24 h and then washed with serum-free media. Cells were then incubated with DCFDA (20 μM) at 37°C for 30 min in the dark prior to washing and manufacturer's buffer in preparation for analysis. Fluorescence intensity was determined by fluorescence spectroscopy with maximum excitation and emission spectra of 495 and 529 nm, respectively.

### Measurement of mitochondrial membrane potential

Mitochondrial transmembrane potential (Δ*Ψ*_m_) in T84 and COLO 205 cells was measured using a tetramethylrhodamine ethyl ester (TMRE) mitochondrial membrane potential assay kit (#ab113852, Abcam) following the manufacturer's instructions. Briefly, 2 × 10^4^ T84 and COLO 205 cells/well in a 96-well plate were challenged with the indicated dose of Andro for 24 and 48 hrs, then incubated with 500 nM TMRE for 20 mins. The media was then gently removed and replaced with 100 μl of PBS/0.2% BSA. This step was repeated twice to remove excess dye. The fluorescence signal was quantified with Ex/Em = 549/575 nm.

### SDS-PAGE and western blot

SDS-PAGE and western blotting were performed as previously described [18, 45]. The protein content for samples was determined using the BCA Protein Assay Kit (Pierce). Samples containing approximately 20–40 μg of protein were boiled in LDS sample buffer, separated on SDS-polyacrylamide gels, electrophoretically transferred to Immunoblot-P transfer membranes (IPV H00010, EMD Millipore corporation), and probed with the following primary antibodies; IRE-1 (#3294S), p-AKt (Ser 473) (#9271), AKt (#9272), pAKt (Thr 308) (#9275S), GPX (#3206S) from Cell Signaling Technology (Danvers, MA), Cyclin B1 (GNS1, #sc-245), cyclin D1 (#sc-8396), cyclin A (#H-432), PRx (A-6) P (Sc-137150P) from Santa Cruz Biotechnology (Dallas, TX), and GAPDH (#G8795) from Sigma Aldrich. The proteins were visualized using horseradish-peroxidase-conjugated secondary antibodies (#7074, Cell Signaling Technology) followed by Supersignal West Pico Chemiluminescent Substrate (#34087, Thermo Scientific). Results were quantified by densitometry of digitized images using ImageJ software (NIH, Bethesda, MD, ver.1.43) and expressed as a ratio to a GAPDH loading control.

### Quantitative real-time polymerase chain reaction (qRT-PCR)

Gene expression was evaluated as previously described [46]. Total RNA was purified using the EasyPure^®^ RNA kit from Annagen Biotech (Baltimore, MD) or the High Pure RNA Isolation Kit from Roche Life Science (Indianapolis, IN) The cDNA prepared with TransScript^®^ First Strand cDNA Synthesis Supermix (Annagen Biotech) or First Strand cDNA Synthesis Kit for RT-PCR (Roche Life Science). PCR amplification was performed with an Eppendorf Realplex Instrument (Eppendorf AG, Hamburg, Germany) with SYBR Green supermix (Fermentas, Glen Burnie, MD), 0.8 μM of each primer, and 1 μl cDNA. Primer sequences are listed in Supplementary Table 1. Relative gene expression changes were calculated using the 2^−ΔΔCT^ method, and expression normalization was accomplished using housekeeping gene glyceraldehydes 3-phosphate dehydrogenase (GAPDH).

### Measurement of Caspase -3 activity

Caspase-3 activity was determined by colorimetric assay according to manufacturer's instructions (#APT165, EMD Millipore, Billerica, MA). Cells (1 × 10^6^) were treated with Andro and NAC at the desired concentrations were incubated for 24 h and 48 h. Cells were then harvested from the plates and washed by centrifuged at 1500 rpm for 10 min. Cells were resuspended in 100 μl of chilled 1X cell lysis buffer and incubated on ice for 10 minutes. The cytosolic extract was recovered after centrifugation for 5 mins at 10,000 g. Samples were mixed with assay buffer and caspase 3 substrate and then incubated for 2 h at 37°C. Absorbance (OD_405 nm_) was determined using a ThermoMax plate reader (Molecular Devices, Sunnyvale, CA)

### Fluorescence microscopy and image acquisition

Immunofluorescence was performed as previously described [47]. Briefly, cells were grown on glass cover slips for 24 h and treated with or without Andro and NAC as indicated. Cells were then fixed in ice-cold methanol for 5 min, permeabilized with 0.2% Triton X-100 for 10 min, blocked with 6% BSA for 30 min and then incubated with a 1:50 dilution of phospho-mTOR (Ser 2481) (#2974, from Cell Signaling Technology), or TRX (#sc-271281 from Santa Cruz), overnight at 4°C. Cells were then incubated with Alexa Flour 594 labeled goat anti-rabbit IgG (#A11037); or Alexa Flour 488 (**#**: A-11001) from Thermo Fisher Scientific at 1:200 and DAPI for 1 h [18]. Sample images were taken at 200 × magnification using an inverted fluorescence microscope (Olympus IX-7, Pennsylvania, USA Fluorescence intensity was quantified using ImageJ software version 1.39 (NIH). RGB composite images were created using Axion Vision rel, 4.6 and analyzed. Cells from five different fields were used for statistical analysis as previously described.

### Statistical analysis

Data are presented as mean ± standard Error (S.E). Statistical analysis was carried out with Graph Pad Prism for Macintosh 5.0c (Graph Pad Software Inc., San Diego, CA). The mean S.E. was calculated by one-way analysis of variance (ANOVA). Significance between groups was further analyzed using the post hoc Tukey's test and Bonferroni test. *P* values were considered significant is less than 0.05 and are indicated as throughout using asterisks *P < 0.05, **P < 0.01, ***P < 0.001.

## SUPPLEMENTARY MATERIALS FIGURES AND TABLES


