# Supplementary material for: Increased reactive oxygen species levels cause ER stress and cytotoxicity in andrographolide treated colon cancer cells
Source: Oncotarget. 2017 Feb 16;8(16):26142–53. doi: 10.18632/oncotarget.15393 (PMC5432246; doi:10.18632/oncotarget.15393)
Supplement: Supplementary file 1 [file oncotarget-08-26142-s001.pdf]

Increased reactive oxygen species levels cause ER stress and cytotoxicity in andrographolide treated colon cancer cells

Supplementary Materials

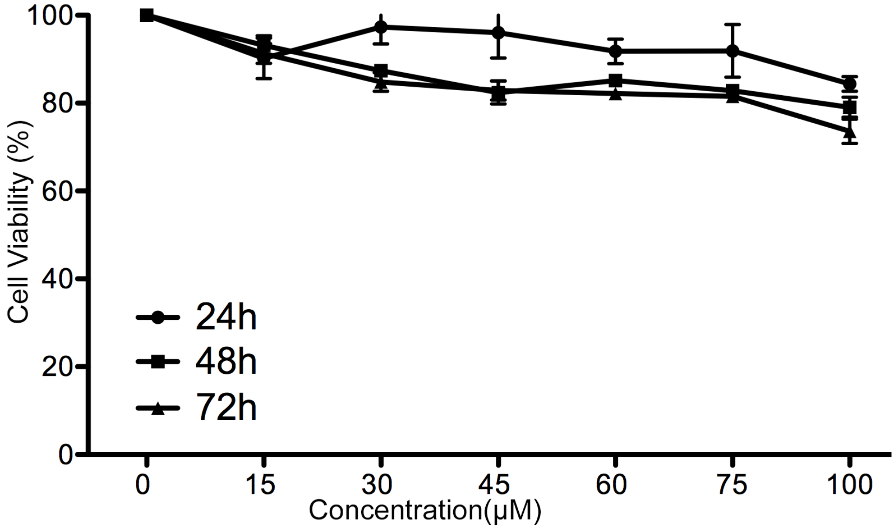

Supplementary Figure 1: Andrographolide effects on normal colon epithelial cells FHC. Normal colon epithelial cells were treated with the recommended complete medium containing a final concentration of 0.01% DMSO. The FHC cells were treated with 0, 15, 30, 45, 60, 75, 100 μM Andro for 24 h, 48 h and 72 h.

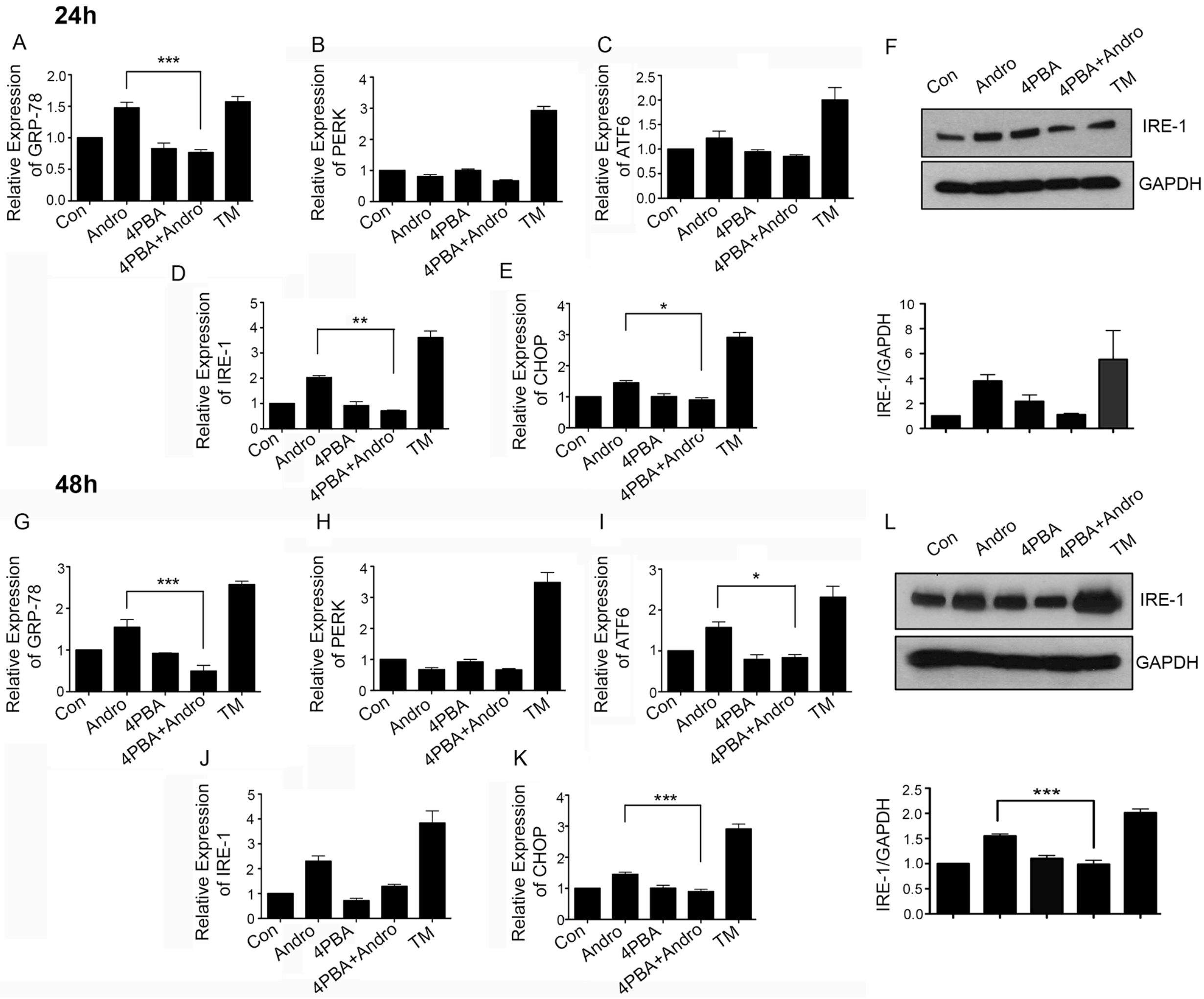

Supplementary Figure 2: Andrographolide induces ER stress-related IRE-1 and associated proteins in COLO 205 cells. (**A**) COLO 205 cells were treated with Andro IC_{50} in the presence or absence of 4-PBA for 24 h and 48 h and the transcriptional level of expression for ER stress and apoptosis associated genes was determined by qRT-PCR for A, (**G**) GRP-78, (**B**, **H**) PERK, (**C**, **I**) ATF6, (**D**, **J**) IRE-1 and (**E**, **K**) CHOP. Bar graphs show quantitative results normalized to GAPDH mRNA levels. Results are from three independent experiments. Statistical significance was determined using one way-ANOVA followed by post hoc Tukey's test. (*$P < 0.05$, ** $P < 0.01$, *** $P < 0.001$). Protein expression was determined for IRE-1 at (**F**) 24 h. and L. 48 h. and quantified by densitometry. Tunicamycin (TM) at 2 µg/ml was used as a positive control. The results shown are from three independent experiments (***$P < 0.001$).

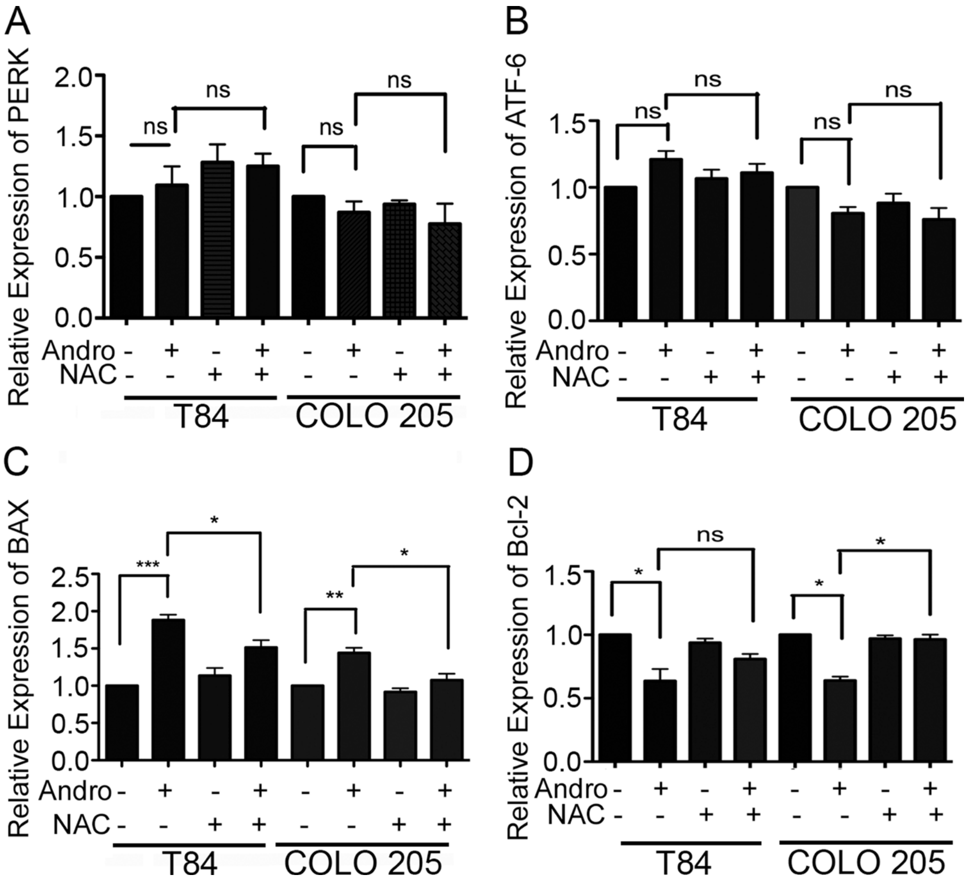

Supplementary Figure 3: Andrographolide induces ROS dependent apoptosis signaling. Total mRNA from T84 and COLO 205 cells pretreated with or without NAC followed by treatment with Andro IC_{50} were subjected to qRT-PCR for the expression of (**A**) PERK, (**B**) ATF-6, (**C**) BAX, and (**D**) Bcl-2. The results shown are from three independent experiments (ns = non-significant, **P* < 0.05, ***P* < 0.01, ****P* < 0.001).

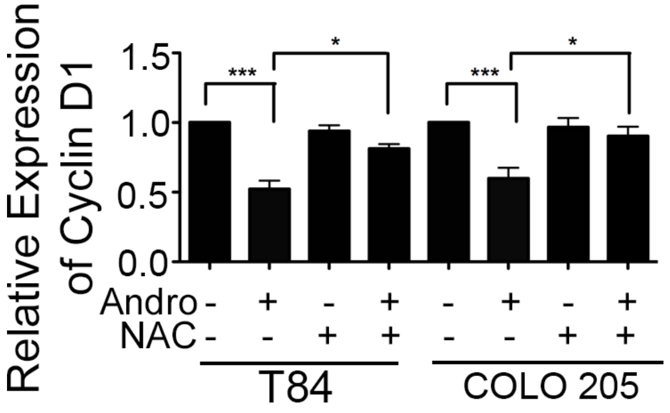

Supplementary Figure 4: Andrographolide induces ROS dependent cell cycle arrest at G1/S phase. Total mRNA from T84 and COLO 205 cells pretreated with or without NAC followed by treatment with Andro IC_{50} were subjected to qRT-PCR for the expression of Cyclin D1. The results shown are from three independent experiments (**P* < 0.05, ****P* < 0.001).

Supplementary Tabe 1: qRT-PCR primers

Gene	Primer sequence forward	Primer sequence reverse
Gene	Primer Sequence Forward	Primer Sequence Reverse
GRP-78	5′-GGTGAAAGACCCCTGACAAA-3′	5′-GTCAGGCGATTCTGGTCATT-3′
IRE-1	5′-GGGAAATACTCTACCAGCCT-3′	5′-GAAATCTCTCCAGCATCTTG-3′
PERK	5′-ATCCCCCATGGAACGACCTG-3′	5′-ACCCGCCAGGGACAAAAATG-3′
ATF6	5′-TCAGGGAGTGAGCTACAAGT-3′	5′-CTTGTGGTCTTGTTATGGGT-3′
CHOP	5′-TTCTCTGGCTTGGCTGACTG-3′	5′-CTGCGTATGTGGGATTGAGG-3′
LPO	5′-GGATGCCAGCTTTGTGTAC-3′	5′-TAGGGCAGGTAGGGTAGTC-3′
Nrf-2	5′-CAGCGACGGAAAGAGTATGA-3′	5′-TGGGCAACCTGGGAGTAG-3′
PrX-1	5′-ATGTCTTCAGGAAATGCTAAAAT-3′	5′-TCACTTCTGCTTGGAGAAATATTC-3′
PrX-6	5′-GGACGTGGCTCCCAACTTT-3′	5′-CGAGGGTGGGAGAAGAGAATG-3′
TRX	5′-TGAAGCAGATCGAGAGCAAGAC-3′	5′-TTCATTAATGGRGGCRRCAAGC-3′
GPX-2	5′-GGCTTTCATTGCCAAGTCCTTC-3′	5′-CTATATGGCAACTTTAAGGAGGCGC-3′
Cyclin D1	5′-CACACGGACTACAGGGGAGT-3′	5′-AGGAAGCGGTCCAGGTAGTT-3′
GAPDH	5′-CGACCACTTTGTCAAGCTCA-3′	5′-AGGGGAGATTCAGTGTGGTG-3′
Bax	5′-GAGAGGTCTTTTTCCGAGTGG-3′	5′-CCTTGAGCACCAGTTTGCTG-3′
Bcl-2	5′-GGAGGCTGGGATGCCTTT3′	5′-ACCCATGGCGGTGACCATGC-3′
